# Integrating Gait Analysis and AI Into Knee Osteoarthritis Care: Protocol for a 3-Phase Participatory Qualitative Study

**DOI:** 10.2196/82860

**Published:** 2026-04-13

**Authors:** Owen Ryan Lindsay, Janie L Astephen Wilson

**Affiliations:** 1 School of Biomedical Engineering, Department of Surgery, Faculties of Engineering and Medicine, Dalhousie University, Dentistry Bldg, 5th Fl., 5981 University Avenue, Halifax, NS, B3H 4R2, Canada, 1 4038089709

**Keywords:** knee osteoarthritis, arthroplasty, clinical decision support, artificial intelligence, AI, qualitative research, gait analysis, patient participation, interview, user-centered design, implementation

## Abstract

**Background:**

Knee osteoarthritis (OA) leads to pain, disability, and reduced quality of life. For advanced stages, knee arthroplasty surgery is the standard treatment, yet dissatisfaction and persistent mobility deficits remain common. Current surgical decision-making processes seldom incorporate objective predictors of outcomes, such as biomechanical data. Advances in computer vision, wearable sensors, and artificial intelligence now enable efficient capture and interpretation of clinically prognostic gait features in real-world settings. However, the clinical adoption of such innovations remains limited, hindered by usability challenges, misalignment with stakeholder needs, and system-level barriers.

**Objective:**

This study aims to inform the development of a knee OA clinical decision support (CDS) tool that integrates gait analysis and artificial intelligence with clinical decision-making. More broadly, it seeks to advance a framework for participatory digital health CDS tool implementation by identifying and addressing suboptimal workflows, stakeholder priorities, and organizational challenges.

**Methods:**

This study uses a 3-phase participatory design process, with in-depth interviews conducted in each phase to generate insights that inform subsequent stages. Phase 1 investigates perceived barriers to and facilitators of the implementation of a digital CDS tool and examines current workflow processes in knee OA management. Phase 2 focuses on identifying and defining user requirements to guide solution ideation for translational digital decision support. Phase 3 assesses user acceptance of prototyped solutions. Field observations in phase 1 and usability testing in phase 3 will supplement interview findings with real-world evidence.

**Results:**

The study was funded in November 2024. Ethics approval was granted by the Nova Scotia Health Research Ethics Board on July 28, 2025. Data collection began in September 2025. As of February 2026, a total of 6 clinicians have been interviewed, with all 6 interviews transcribed and 4 coded as part of phase 1 analysis. Study completion is anticipated by August 2026. Findings will be published by January 2027.

**Conclusions:**

Our stakeholder-driven approach prioritizes both technological rigor and practical usability, addressing key human factors for successful implementation. While initially focused on Nova Scotia’s orthopedic setting, this research provides a scalable framework for integrating digital decision support tools into broader clinical environments, advancing innovation in knee OA care and beyond.

## Introduction

### Background

Knee osteoarthritis (OA) is a progressive and debilitating disease characterized by pain, disability, and poor health-related quality of life, with its incidence on the rise. For advanced stages, knee arthroplasty surgery is the standard treatment, intended to relieve symptoms and restore function and joint mobility. Despite the common use of arthroplasty, the spectrum of relevant and multifaceted [1] knee arthroplasty surgery outcomes is not universally positive [1-5]. Approximately 1 in 5 patients is not satisfied after surgery [5], and upward of 70% experience continued walking deficits years after surgery [2]. These mixed results reflect the fact that treatment decisions are often based on limited indicators, such as pain severity or radiographic changes, rather than a multidimensional understanding of the patient. Evidence shows that combinations of factors, including pain, gait function, joint structures, and patient expectations, better predict recovery [6,7], yet these predictors are rarely integrated systematically into clinical workflows.

Objective measures of function, particularly gait analysis outcomes, provide critical insights into OA progression and surgical response [8,9]. Deficits in knee joint mechanics are strongly linked to patient-reported pain, functional outcomes [7], and even implant migration and early failure [10-12] following knee arthroplasty surgery. However, traditional gait analysis has required costly, complex laboratory systems, limiting uptake in clinical practice. Recent advances in computer vision, wearable sensors, and machine learning now make it feasible to capture prognostic gait features more readily in real-world clinical environments [13-16]. Broad investment in advanced computing and artificial intelligence (AI) augments the potential of such systems, enabling the integration of biomechanical biomarkers with patient-reported outcome measures, activity levels, joint morphometrics, and demographics for clinically meaningful, individualized risk predictions and decision support [17].

A major gap in translating these technological advances into practice lies in implementation. Despite their promise, clinical decision support (CDS) tools of this nature seldom progress beyond the research stage due to poor usability, lack of alignment with clinician and patient needs, and organizational constraints that hinder adoption [18,19]. These challenges highlight the importance of early, systematic engagement with stakeholders to ensure that CDS tools are designed with contextual, organizational, and human factors in mind.

This protocol describes a participatory qualitative study to inform the development of a multidimensional knee OA decision support tool. Guided by implementation science [20] and design thinking principles, the study will iteratively investigate clinical workflows (phase 1), specify stakeholder values and user requirements (phase 2), and test early prototypes for usability and acceptance (phase 3). By situating technical innovation within a stakeholder-driven design process, this study aims to inform how to bridge the persistent gap between digital health potential and real-world clinical adoption for knee OA management.

### Objectives

Our long-term goal is to develop an objective, evidence-driven decision support tool that promotes consistent, enhanced patient outcomes both during the surgical wait period and after knee arthroplasty surgery. This protocol outlines the qualitative methods guiding the participatory design and development of a CDS tool prototype, which will form the foundation for future research into clinical implementation.

#### Phase 1: Contextual Inquiry

The aim of phase 1 is to understand and map workflows comprising surgical evaluations, follow-up visits, and decision-making processes for knee arthroplasty surgery candidates; assess how clinicians communicate risks to patients and determine appropriateness for surgery; elucidate potential challenges or barriers to integrating gait analysis and AI-enabled CDS tools into routine clinical practice; and identify relevant stakeholders, including interdisciplinary clinical team members, patients, and decision-makers, who may further inform the development and implementation of the tool.

#### Phase 2: Value Specification

The aim of phase 2 is to identify and characterize touchpoints within clinical workflows and across the perioperative period where a digital support tool would provide optimal value to users; refine features using stakeholder insights, ensuring that multidimensional patient data (eg, gait biomarkers, patient-reported outcome measures, activity levels, morphometrics, and demographics) are translated into actionable, transparent risk predictions; and assess the role of CDS and AI on patient-clinician communication and decision-making, including users’ willingness to engage with a tool and its specific components.

#### Phase 3: Design

The aim of phase 3 is to design a functioning CDS system prototype; assess the tool’s intuitive usability by evaluating interface navigation, data visualization, and integration into clinical workflows; and identify user-perceived benefits, barriers, and areas for improvement, thereby informing translational and iterative refinements to tool development.

## Methods

### Study Design

Drawing from literature on technology adoption and sustainability in health care [21,22], this protocol deliberately moves beyond the typical solution-driven approach, whereby system intelligence and technical capacity are the primary focus of design. Instead, it grounds these components in human experiences, offering a more empathetic approach that balances system capabilities with diverse stakeholder perspectives. In the context of implementation, where stakeholder perspectives must span many levels of health care, it is necessary that methodologies be designed to capture, organize, and integrate inputs from multidisciplinary team members and end users with varying roles and levels of expertise.

To accomplish this, our protocol follows a holistic framework guided by the Centre for eHealth and Wellbeing Research (CeHRes) Roadmap [20] and enriched by principles of design thinking [23]. Our methodologies expand on the CeHRes Roadmap by incorporating complementary theoretical constructs known to influence implementation success, scalability, and sustainability, including organizational readiness, health literacy, and contextual resilience [21]. This protocol is grounded in three core principles derived from these frameworks: (1) active engagement and oversight by a multidisciplinary team of key opinion leaders, clinicians, designers, and engineers; (2) iterative evaluation cycles with user-driven feedback loops to ensure that tool functionality reflects the lived experiences and expertise of unique stakeholder groups; and (3) ongoing consideration of adoption and scalability across all stages of research to support future uptake.

### Setting and Participants

In phase 1, we will recruit orthopedic surgeons responsible for evaluating patient appropriateness for knee arthroplasty surgery. These clinicians will participate in individual interviews conducted either at their clinic site or remotely for convenience. They may also optionally participate in field observations of clinical consultations, which will be conducted at their clinic site. A purposive sampling strategy will be used to recruit participants across a range of experience levels, from surgical fellows to senior clinicians. Clinicians and fellows who are not directly involved in perioperative care or decision-making for knee OA will be excluded.

Building on phase 1 outcomes, phase 2 will use respondent-driven sampling to re-engage previous participants and expand recruitment to additional stakeholders whose perspectives are relevant to tool development. Eligible participants will include allied health professionals, health care administrators, and policy-level decision-makers involved in clinical workflow design or technology adoption, whereas industry representatives or individuals not directly connected to clinical implementation will be excluded. Additionally, patients with clinical diagnoses of knee OA who are being evaluated or scheduled for knee arthroplasty surgery will be recruited during OA clinic visits, and those with prior knee arthroplasty surgery may also be included if they are awaiting revision surgery or undergoing surgery on the contralateral limb. Participants from phase 2 will be invited to continue into phase 3, with supplementary recruitment as necessary. To minimize travel burdens, interviews and usability testing in these phases will be offered both in person at participants’ workplaces and virtually using videoconferencing software when appropriate.

### Ethical Considerations

This study was reviewed and approved by the Nova Scotia Health Research Ethics Board (file 1031703; approval date: July 28, 2025). Eligible patients will first be asked for verbal consent to be contacted by a co–principal investigator during a clinical encounter and, if interested, will complete written informed consent electronically via REDCap (Research Electronic Data Capture; Vanderbilt University) prior to participation. Clinical staff and decision-makers will be recruited through email invitations and will similarly provide written informed consent before participation. All identifying information will be used solely for scheduling and coordination and will be stored securely in accordance with institutional privacy and confidentiality protocols. Study data will be deidentified prior to analysis. No financial compensation or other incentives will be provided to participants for their involvement in this study.

### Sample Size

Estimating sample sizes in qualitative research a priori is inherently challenging due to its evolving and emergent nature. For health care research, Hamilton and Finley [22] advise recruitment of 5 to 10 participants in key roles within a single health care setting. However, saturation often extends beyond this range [24-28]. Acknowledging the role of methodologies, researcher expertise, study objectives, and other factors in reaching saturation, Mthuli et al [29] developed the “define, explain, justify, apply” tool, which has been adopted as our guiding framework:

- Define—a nonprobability purposive sampling strategy will be used to capture context-rich insights. Initially, we will recruit orthopedic surgeons responsible for knee arthroplasty surgery decisions. In subsequent phases, respondent-driven sampling will broaden our participant pool to include patients, other care team members, and decision-makers.
- Explain—this sampling strategy aligns with our aim of deeply understanding real-world clinical workflows and stakeholder experiences, ensuring that early phases focus on clinician insights whereas later phases incorporate diverse perspectives.
- Justify—prioritizing depth over breadth is critical in exploratory qualitative research. Focusing first on clinicians secures alignment with clinical practices, and expanding the sample later enhances the tool’s relevance across various health care contexts.
- Apply—in phase 1, we aim for 5 to 10 participants to gather in-depth, context-rich insights from key clinical stakeholders. In phase 2, this pool will be expanded to 15 to 30 participants across multiple user groups (ie, clinicians, allied health professionals, administrators, organizational decision-makers, and patients), incorporating a broader range of perspectives. Finally, phase 3 will involve iterative evaluations with 5 to 10 participants per primary user group, who will provide ongoing feedback in response to prototypes of increasing fidelity (estimate of 3 iterations). These sample sizes are consistent with previous research addressing contextual inquiry [25,30], value specification [31-33], and design objectives [34-36].

### Research Phases

#### Overview

**Figure 1.**
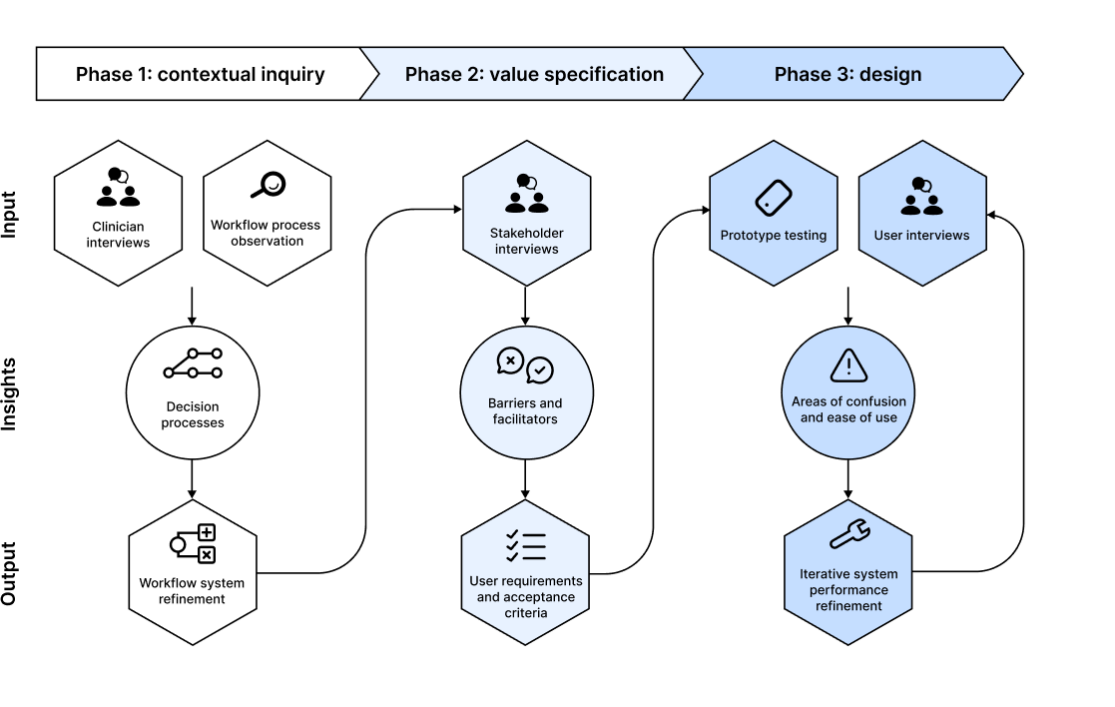
Schematic representation of the 3 phases comprising preliminary clinical decision support tool development.

Our research uses in-depth qualitative inquiry to investigate the tool’s intended environment and user groups (Figure 1). Specifically, we aim to understand perceived barriers and facilitators, current workflow processes (phase 1), user requirement identification and definition (phase 2), and prototype functionality (phase 3). The methods are designed to systematically build on each phase: phase 1 identifies workflow barriers, phase 2 expands on these barriers and reframes them as design opportunities, and phase 3 tests solutions to these barriers through prototypes. Methods include one-on-one interviews, field observations, and think-aloud usability testing, all of which are well suited to achieve these objectives based on the research team’s prior experience.

#### Phase 1: Contextual Inquiry

The first phase of this study involves formative research to understand process workflows throughout the perioperative patient journey and during clinic visits. The CeHRes Roadmap toolkit acknowledges that the very development of health care technologies creates new processes within health care delivery [19]. Understanding these processes is critical as previous studies have shown that technology abandonment may result from disrupted workflows, changes in staff roles, and a low organizational capacity to support technology innovation [18,21]. Semistructured interviews comprising open-ended questions will explore the process for determining appropriateness for knee arthroplasty surgery, including which decision-making resources and information are used to determine appropriateness and timing for surgery (Multimedia Appendix 1). Clinicians will also be given the opportunity to provide insights into perceived anticipated challenges or barriers to the digital CDS concept, including organizational readiness, workflow fit, and capacity to support implementation and sustained use.

To corroborate interview data [37] and ensure that tool development is grounded in a real-world clinical context, participating clinicians may optionally consent to have a research member conduct observations of patient consultations. Prior to each observed consultation, clinicians will inform patients that a researcher will be present to observe clinic workflow and will request verbal consent for the researcher to remain. Patients may decline without impact on their care. Field notes will document only workflow elements (eg, sequence of tasks, decision points, information sources, and physical environment) [38] and will exclude all patient-identifiable information and clinically sensitive details. Both clinicians and patients may request the researcher’s departure at any time, and interactions with patients will be limited during observations to avoid disrupting clinical workflows.

Direct observations will allow us to capture unspoken processes, contextual factors, and decision-making dynamics that may not emerge in verbal accounts. Additionally, field notes will serve as a reference to map workflow processes by capturing inputs, outputs, and steps of decision-making as they unfold in real time [38,39]. Where discrepancies between interview-reported and researcher-observed workflows emerge, these will be explored through follow-up member checking to understand underlying reasons (eg, situational variation, informal workarounds, and idealized vs actual practice) as such gaps may reveal implementation barriers and design requirements for workflow variability.

Phase 1 will conclude when contextual understanding of current workflows, decision-making processes, and implementation barriers has reached thematic saturation, yielding validated workflow maps and a barrier framework to inform further inquiry into user values and requirements.

#### Phase 2: Value Specification

Building on the findings from the contextual inquiry, this phase will further explore user requirements to drive solution ideation. Using an inductive approach, we will analyze themes from phase 1 to identify the high-level barriers and challenges anticipated by stakeholders. These insights will guide a deeper investigation into specific opportunities for development, helping us prioritize user requirements based on their values.

Specifically, we will explore how technologies such as motion capture, AI, system integrations, and supporting devices (eg, video cameras, wearable sensors, desktop computers, smartphones, and tablets) relate to identified barriers. Understanding how users ideally wish to interact with these technologies, as well as their preferences for how the system should generate, translate, and present knowledge for decision-making, is essential for defining system inputs and establishing benchmark usability requirements. Additionally, interviews will explore deployment material needs, including training resources, workflow integration guides, and technical support requirements necessary for successful adoption. Insights from these interviews will be used to align tool development with previously identified barriers, informing both user needs definition and feature acceptance criteria [21,40]. Phase 2 will conclude when consensus on user-defined priority features and deployment needs has been reached, providing the specifications necessary to guide prototype development in phase 3. Because the value specification process builds on previous findings, the interview guide will be developed after completing phase 1 analysis to ensure alignment with emerging themes.

#### Phase 3: Design

The design cycle focuses on translating identified user needs and operational requirements into a refined prototype and deployment materials. Prototypes will be interactive to support evaluation of user experiences. For usability testing, participants will be provided with training and deployment materials developed based on earlier-phase evidence and asked to complete hypothetical, user-specific scenarios using the CDS system prototype while verbalizing their thought processes. Following prototype interaction, participants will be asked brief follow-up questions to capture overall impressions and suggestions for improvement.

Interactive prototypes will display synthetic, representative patient data to generate simulated patient-specific risk profiles. These outputs will be visualized in case report format in a digital user interface, presenting users with realistic (but synthetic) patient scenarios. It is important to note that this study does not serve to test model accuracy, validate predictive performance, or make effectiveness claims regarding clinical outcomes. Rather, it explores users’ behavioral responses to model outputs and visualizations in the context of clinical decision-making, identifying design requirements and implementation barriers prior to formal validation studies.

Usability evidence generated through phase 3 will inform refinement of the CDS tool prototype and deployment protocols, positioning these outputs for subsequent validation studies and implementation planning. Future research will assess the tool’s impact on surgical decision-making, patient outcomes, and health care resource use.

### Analysis

#### Interviews and Think-Aloud Testing

Interview sessions and think-aloud testing will be audio recorded and transcribed verbatim, with transcripts verified against recordings by a second research team member for accuracy. In each phase, the 2 researchers (ORL and JLAW) will review and analyze transcripts via NVivo (version 14.24.3; Lumivero) using an inductive thematic approach without prior theoretical influences [41].

The researchers will independently code early transcripts to generate initial inductive codes, bringing their unique disciplinary backgrounds to the interpretation. For each coding decision, the researchers will document their rationale in reflexive memos, creating an audit trail that captures the reasoning behind interpretive choices. Codes and associated memos will be shared at regular meetings to discuss coding decisions, examine how the researchers’ perspectives on AI and clinical decision-making may influence interpretation, and explore whether coding discrepancies reflect genuine ambiguity in the data or researcher assumptions. Discrepancies will be treated as opportunities to surface alternative perspectives, refine understanding, and mitigate individual bias.

Through this iterative process, codes will be expanded, collapsed, or modified as new transcripts are analyzed, with ongoing documentation of analytical decisions and their relationship to emerging vs preexisting beliefs about CDS tool implementation. Previously coded transcripts will be revisited as needed when the coding framework evolves. Data collection and analysis will proceed concurrently to allow for the full exploration of emerging themes. This iterative and reflexive approach to thematic analysis aligns with the methodology described by Braun and Clarke [41]. Sampling will continue until inductive thematic saturation is reached, meaning that no new themes or codes are expected to emerge from further data [42].

Analysis of usability data will explore user preferences regarding data interpretability, information hierarchy, and communication approaches. The role of this tool in clinical decision-making will be assessed by how clinicians interpret and integrate prognostic information into clinical reasoning and the perceived utility of this information for triage and treatment decisions. Usability findings will be interpreted in conjunction with contextual evidence gathered in phases 1 and 2, such as participants’ roles, organizational constraints (workflow constraints and technical infrastructure), and individual characteristics (health literacy and technical proficiency) to enrich our interpretation of user interactions.

No formal quantitative usability analysis or hypothesis testing will be conducted. Task completion times will be noted during usability testing sessions as contextual information to support the interpretation of qualitative findings.

#### Process Mapping

Realistic accounts of field observations during clinical consultations will be recorded as they occur (ie, in situ) [38]. A deductive framework will guide the initial classification of workflow components (eg, inputs, decisions, and outputs), whereas inductive coding will capture emergent factors that extend beyond process mapping but are still relevant to implementation. To construct a process map, sequences of events comprising clinical workflows will be charted chronologically, identifying points where a CDS tool could integrate into and support existing workflows.

### Quality and Rigor

Ensuring rigor in qualitative research requires strategies aligned with the study’s methodological approach and objectives [43]. Accordingly, this study integrates measures to enhance validity, reliability, and rigor across all stages of data collection and analysis.

To enhance data richness, interview guides will first be pilot-tested with participant representatives, including clinicians, patients, and organizational decision-makers, to refine question clarity, relevance, and reliability prior to data collection [22]. Following pilot-testing, the guides will remain flexible and may be iteratively adapted during data collection in response to emerging themes. Insights generated during the contextual inquiry phase will also inform subsequent sampling strategies, ensuring that the study remains responsive to participants and to data not initially anticipated within the tool’s scope [44]. This adaptive approach will be combined with recruitment strategies designed to promote continuous participant engagement across compounding phases, enabling continuous checking that the data are contextually grounded.

To enhance trustworthiness, real-time member checking will be used during interviews, wherein participant responses will be summarized or restated to confirm accuracy and encourage clarification or elaboration [43]. Given the lack of standardized guidelines for modifying data following transcription or analyses, full transcripts or completed analyses will not be returned to participants for validation [44].

Triangulation will occur at multiple stages to verify data accuracy. Inductive thematic analysis will include ongoing comparison, with independently generated codes shared between the 2 researchers to identify discrepancies, examine interpretive assumptions, and iteratively refine the analytical framework. Additionally, triangulating interview data with direct observations of clinician workflows will help corroborate the findings and identify discrepancies between reported and observed practices (eg, clinician-reported vs researcher-observed workflows) [45].

## Results

This study received funding in November 2024. Recruitment and pilot-testing of study procedures commenced in September 2025. As of February 2026, a total of 6 clinicians have been interviewed, with all 6 interviews transcribed and 4 coded as part of phase 1 analysis. Data collection is expected to continue until August 2026, with findings from each phase feeding into iterative design cycles. The results from this study will be synthesized to inform a framework for integrated CDS technologies in orthopedics and are anticipated to be disseminated in peer-reviewed publications and conference presentations beginning in January 2027.

## Discussion

### Expected Findings

This study protocol describes a participatory, multiphase qualitative approach to developing a digital CDS tool incorporating gait technologies to support clinical decisions for advanced knee OA guided by the CeHRes Roadmap and design thinking principles. By centering the research process on implementation, our methodologies actively challenge designer bias and prior assumptions, incorporating real-world insights into clinical workflows and stakeholder experiences to validate anticipated barriers and uncover unforeseen challenges to adoption and sustainability. In doing so, our approach extends beyond technical development and directly addresses why CDS tools often fail to achieve adoption or sustainability.

Implementing decision support technologies is an inherently delicate process as they introduce workflows and ideologies that may potentially disrupt established health care ecosystems [46,47]. If executed poorly, this can challenge the longevity of CDS tools, possibly resulting in nonadoption, abandonment, or limited scalability [21]. Long-term viability hinges on tools that effectively recognize and balance diverse personal and operational factors [48], usually through design that acknowledges both typical and edge use cases [49,50]. Our approach to this design challenge is to engage clinicians, patients, health care leaders, and decision-makers from the outset, continuously checking that prototype development meets the distinct goals of these user groups at critical decision points in the development process [51].

Research aimed at advancing health care technologies operates in a challenging space where rapid innovation must be balanced with rigorous, evidence-based methodologies. From a methodological perspective, this study contributes to the broader digital health field by demonstrating how participatory design can be operationalized in the context of CDS tool development. Traditional linear models of health technology design, where design, development, testing, and deployment occur in distinct and sequential stages, provide a logical structure but are poorly suited to the rapid iteration required for digital health innovation [52]. Our 3-phase design introduces structured feedback loops that allow evidence from interviews, observations, and usability testing to directly inform functional requirements and software prototypes. This agile framework combines the rigor of qualitative research with the flexibility required for real-world translation, offering a replicable model for testing other CDS technologies where implementation has proved challenging.

In the context of knee OA, this research also carries important clinical implications. A digital CDS tool that incorporates gait biomarkers has the potential to substantially improve both patient- and system-level outcomes. By providing clinicians and patients with objective, individualized prognostic information, the tool could help optimize surgical timing, reduce inappropriate or premature procedures, and support monitoring in the postoperative period. At the health system level, gait-informed CDS may streamline triage, reduce unnecessary clinic visits, and improve resource allocation while also generating large-scale datasets to advance research on OA progression. Future validation studies and implementation planning will build on the outcomes of this study to assess the tool’s impact on surgical decision-making, patient outcomes, and health care resource use.

### Limitations

While these methods were chosen to enhance the overall quality of this study, we acknowledge the limitation that this research is focused specifically on an orthopedic setting in Nova Scotia in Canada’s health care system, which is a specific ecosystem within a publicly funded, resource-constrained environment. This targeted approach enables a deep, context-specific analysis and a tailored implementation strategy but may limit the generalizability of our findings to other health care environments. However, we view this as a necessary first step in establishing a robust, small-scale implementation model that can later be adapted and scaled to more diverse settings.

### Conclusions

This protocol sets out an approach to developing a knee OA decision support tool that is tethered to implementation, combining stakeholder perspectives, participatory design, and iterative refinement. Rather than treating technology development and adoption as separate challenges, our methodologies integrate them into a single process, ensuring that translational tools evolve in step with the needs and realities of their intended users. Although initially situated within an orthopedic context in Nova Scotia, this work represents an important step toward leveraging digital innovation to improve decision-making and patient outcomes in OA care while also offering transferable insights into how complex health technologies can progress from promising concepts to sustainable tools.

## Supplementary material

10.2196/82860 Multimedia Appendix 1 Phase 1 interview script—clinical integration of gait analysis for decision support.

## Abbreviations

AI: artificial intelligence
CDS: clinical decision support
CeHRes: Centre for eHealth and Wellbeing Research
OA: osteoarthritis
REDCap: Research Electronic Data Capture
