# Supplementary material for: Integrating Gait Analysis and AI Into Knee Osteoarthritis Care: Protocol for a 3-Phase Participatory Qualitative Study
Source: JMIR Res Protoc. 2026 Apr 13;15:e82860. doi: 10.2196/82860 (PMC13075632; doi:10.2196/82860)
Supplement: Multimedia Appendix 1 [file resprot-v15-e82860-s001.docx]

Phase 1 Interview Script – Clinical Integration of Gait Analysis for Decision Support:

**Welcome**

Thanks for agreeing to take part. We appreciate your willingness to participate in our study.

**Introductions**

**Purpose of interview**

Thank you for participating in this discussion. Our aim today is to explore the role of a digital clinical decision-support tool within routine clinical workflows for knee osteoarthritis.

To ensure we capture all the details of our conversation, we would like to record this interview. The recording will be transcribed for analysis by the research team, but the transcription will always be kept confidential. We will delete the recordings once the transcripts are prepared, and we have reviewed the content. Do you consent to us recording the conversation?

Some things you say may be used in a publication with your permission, but all information will be anonymized to protect your identity.

I expect our conversation to last around 30 minutes. If you ever feel tired or wish to stop, please let me know at any time.

**Semi-structured interview guide:**

| Questions | Probes |
| --- | --- |
| 1. Describe what a routine surgical evaluation looks like for a knee replacement candidate with osteoarthritis. | 1. General flow and discussion points? 2. What information/resources are used when determining a patient’s appropriateness as a candidate for surgery. 3. Do you interact with any systems or technology? What are they and when do you use them? 4. What input is considered before versus during a consultation. How do these inputs weigh into a final decision? 5. What about a routine follow-up visit before or after surgery? |
| 1. How are the risks and expected outcomes of surgery communicated to patients? | 1. Does this occur during surgical evaluations? 2. How are they communicated? Verbally or through materials? 3. Are these risks weighed in the decision-making process by yourself or patients? 4. Is information used to inform risk? Does this change on a case-by-case basis? |
| 1. Describe what processes are involved once a patient is deemed appropriate for surgery. | 1. Describe correspondence with patients. Who corresponds with them and at what time points? What about patients who are not deemed appropriate? 2. Beside yourself, what other stakeholders or viewpoints are involved in this care coordination? |
| The next few questions relate to a hypothetical. Imagine there was a tool (like an app) that you could use that would give you a patient's risk of poor outcomes while waiting for surgery and after surgery that included their specific clinical details, images and gait function. | |
| 1. Do you envision any challenges or barriers to implementing such a tool in your current practice? | 1. What aspects of your clinical workflow would be most affected by integrating a tool like this? 2. How likely would you be to use this tool or have an interest in using this tool? 3. Would you prefer a simple risk score, a comprehensive report, or an interactive interface? |
| 1. Is there anything we haven’t touched on that you think might be relevant? |  |
| 1. Are there any other stakeholders or viewpoints we should consult to discuss this hypothetical tool? |  |

**Closing**

Thank you for taking the time to share your insights with us today. Your perspective is invaluable in helping us understand how a clinical decision-support tool might fit into the management of knee osteoarthritis.
